# Comparison of veterinary drugs and veterinary homeopathy: part 2

**DOI:** 10.1136/vr.104279

**Published:** 2017-08-19

**Authors:** P. Lees, L. Pelligand, M. Whiting, D. Chambers, P-L. Toutain, M. L. Whitehead

**Affiliations:** 1 Royal Veterinary College, Hawkshead Campus, Hatfield, Hertfordshire; 2 Hall Manor, Kelly, Lifton, Devon PL16 0HQ, UK; 3 Toxalim, Ecole Nationale Veterinaire de Toulouse, France; 4 Chipping Norton Veterinary Hospital, Banbury Road, Chipping Norton, Oxon OX7 5SY, UK

## Abstract

Part 2 of this narrative review outlines the theoretical and practical bases for assessing the efficacy and effectiveness of conventional medicines and homeopathic products. Known and postulated mechanisms of action are critically reviewed. The evidence for clinical efficacy of products in both categories, in the form of practitioner experience, meta-analysis and systematic reviews of clinical trial results, is discussed. The review also addresses problems and pitfalls in assessing data, and the ethical and negative aspects of pharmacology and homeopathy in veterinary medicine.

## Assessment of efficacy

In medicine and therapeutics, treatments can be evaluated in terms of two differing but complementary scientific frameworks. The empiricist framework assesses whether the treatment actually works in animals or people; that is, it has ‘clinical efficacy’ in the form of a beneficial therapeutic effect measurable in laboratory animals or clinical trials. The reductionist framework assesses how the treatment works – investigating the mechanism of action at a submolecular, molecular, cell, tissue/organ and/or system levels as tested in vitro, ex vivo or in vivo studies – determining whether the treatment exerts effects at these levels that can give rise to a clinical therapeutic effect. A distinction should also be made between clinical ‘efficacy’ and ‘effectiveness’. Efficacy comprises performance of a drug under ideal, controlled circumstances. Effectiveness, on the other hand, constitutes performance under ‘real-world’ conditions. Thus, clinical efficacy answers the question ‘does it work in clinical trials’ while clinical effectiveness addresses the question ‘does it actually benefit patients in practice’ (Godwin and others 2003, Gartlehner and others 2006).

In the whole animal, studies may be conducted in healthy animals, in disease models or in clinical subjects. For every level of testing, controls and statistical analysis should be applied, as appropriate. This approach is the basis of modern science-based medicine. Wherever possible, a product will be tested for clinical efficacy and/or effectiveness in randomised, double-blinded, placebo-controlled trials; these provide the best evidence for the practice of evidence-based veterinary medicine (EBVM), which consists of the application of the best available evidence to practice (Sackett and others 1996). Assessment of clinical efficacy and effectiveness should be based on scientific analysis of data rather than relying on the observations and experiences of practitioners in carrying out their routine duties, because the latter is unreliable (Kohn and others 2000, Shojania 2003, Doust and Del Mar 2004, Hartman 2009, Oxtoby and others 2015, Prasad and Cifu 2015, Saposnik and others 2016).

These approaches have provided good evidence of efficacy and/or effectiveness and/or proven or plausible underlying mechanisms of action for most conventional drug-based products, especially of the more commonly used medicines; particularly in the human field, in which products are tested far more than in veterinary medicine. In contrast, as discussed below, attempts to demonstrate biological, clinically relevant effects of homeopathic products in vitro have not shown any clear successes, and the highest quality clinical trial evidence has failed to show convincing evidence of efficacy of homeopathic remedies (Shang and others 2005, Mathie and Clausen 2014, Mathie and others 2014). From a scientific viewpoint, this is unsurprising given that homeopathic remedies commonly contain little or no ‘active’ ingredient, implying that homeopathic remedies do not exert effects via physiological/biochemical mechanisms that can be scientifically measured.

Yet homeopaths in practice insist their treatments are effective (for example, Kayne 2006, Mathie and others 2007, 2010, Gregory 2008, 2013a, Reilly 2008a, British Association of Homeopathic Veterinary Surgeons 2017, British Homeopathic Association 2017). As discussed in part 1 of this review (Lees and others 2017), homeopathic belief proposes – and, importantly, homeopathic practice implies – that during preparation of a remedy, the ‘active’ ingredient imparts an unknown curative property to the remedy. This is presumably by transference of this property to the diluent or by a transformation of the diluent, because the curative property persists, and is most potent, in highly diluted remedies containing no molecules of the starting substance. This curative property, latent in the starting substance, is made active (‘dynamised’ or ‘potentised’ in homeopathic terminology) by repeated, serial dilution and succussion (a specific type of agitation of the remedy). The more dilution and succussion, the more potent the healing power of the remedy. In the belief system of homeopathy, this curative property is thought to be an ‘energy’, specifically a ‘life energy’ or ‘vital force’ (for example, Kayne 2006, Nicolai 2008, Owen 2015d), that is often described as ‘vibratory’ and ‘resonant’. However, homeopaths are unable to demonstrate this hypothetical ‘energy’ (vide infra). In the belief system of homeopathy, remedies work in a wholly different manner to conventional drugs, frequently stated to consist of an unspecified ‘balancing’ of undefined ‘energies’ (the ‘vital force’) in the body (Bell and others 2004, Kayne 2006). Thus, the fact that there appears to be no scientifically conceivable way in which homeopathic products could act on biochemical pathways or physiologic processes underlying the diseases they treat is not an impediment to their practice. The question to be posed is, can and should homeopathy be evaluated using the same preclinical and clinical methods and standards as for conventional drugs?

## Theoretical and actual bases for efficacy

### Homeopathy

From a scientific, material perspective, homeopathic remedies are physical entities, comprising vast numbers of molecules of diluent – usually water and/or alcohol – with the only other components being: many, few or no molecules of the ‘active’, dependent on the degree of dilution; and any contaminants. Some remedies contain other deliberate additives, such as sugar, but such additives are generally not claimed to contribute directly to the alleged healing effects (Kayne 2006). These liquid remedies may be mixed with or dropped or sprayed onto other pharmaceutical preparations to create homeopathic creams, ointments, pills and powders.

#### Molecules of the ‘active’

Each batch of product, depending on the degree of dilution, might contain many, few or no molecules of the starting substance or ‘active’; for dilutions beyond the Avogadro limit (1x10^-24^, expressed in homeopathic notation as ‘12c’) there should be no molecules of the ‘active’ in the remedy (Kayne 2006). Homeopathic belief and practice implies that for any one remedy, the presence or absence of molecules of the starting substance, or the precise number of such molecules present, is inconsequential for its medicinal effect. This must be the case, because: homeopaths do not usually claim that the starting substances per se can exert the medicinal effects of the ‘potentised’ remedies produced from them; at the lower dilutions – those not beyond the Avogadro limit – the ‘potency’ of the remedy increases with dilution, it is negatively correlated with the number of molecules of ‘active’ present; homeopaths very commonly use remedies diluted beyond the Avogadro limit, and claim that: such ultra-diluted remedies containing no molecules of the starting substance are not only effective, but more effective than less diluted remedies; and that increasing dilution of the remedies increases their effectiveness, even at dilutions where no molecules of the starting substance remain (for example, Hahnemann 2002, Kayne 2006, 2008).

For the great majority of substances from which homeopathic products are made, the starting material per se is not claimed to exert the healing effect of the remedy produced from it. Therefore, the ‘curative property’ must be latent in the starting substance, and is passed into the remedy during the preparation process, starting with – depending on the nature of the substance or thing – dissolving, grinding-up, soaking or percolating it in water and/or alcohol (Kayne 2006). This curative property then has to be activated (‘dynamised’, ‘potentised’ or ‘energised’) by serially diluting and succussing the solution or suspension (the ‘mother tincture’) so obtained (Kayne 2006). Thus, eating onion does not cure a common cold but, homeopaths claim, taking the remedy *Allium cepa*, prepared by grinding up onion in diluent and then serially diluting and succussing the mother tincture until there are few or no molecules of the onion in the remedy, can cure signs and symptoms of a cold (Boericke 2008).

What property of the starting substance brings about the alleged healing effect is not known. However, by simple reasoning, if it is not molecules of the substance, then either some other property of the substance is transferred to the water/alcohol, or the substance must bring about some transformation of the water/alcohol itself. It is that unknown transferred property or transformation of the water/alcohol that must provide the alleged healing effect, once it has been further enhanced with each round of dilution and succussion. As noted above, this unknown property is often referred to by homeopaths as an ‘energy’ and, in homeopathic belief, as manifestation of a ‘vital force’.

Cowan and others (2005) found that water in the liquid state is highly efficient at redistributing its hydrogen bonds with a ‘memory’ of less than 50 femtoseconds (<5x10^-14^ s). Water ‘memory’, of the nature and duration required to comprise a mechanism of action of homeopathic products – to transfer information from the starting substance to the patient who takes the remedy – is not known to physical, chemical and biological sciences. Consistent with this observation, studies of remedies diluted far beyond the Avogadro limit (ie, that would not be expected to contain even one molecule of the ‘active’), including comparisons with control solutions not produced from an ‘active’, have failed to provide any convincing, independently replicated demonstration of any special physical or chemical property of the remedies (Aabel and others 2001, Milgrom and others 2001, Rey 2003, 2007, Roy and others 2005, Elia and others 2006, van Wijk and others 2006, Rao and others 2007, Cartwright 2016). Aabel and others (2001) and Milgrom and others (2001) failed to replicate results from previous nuclear magnetic resonance measurements of homeopathic remedies, and concluded that there was no difference between homeopathic and control solutions. Examples of reported differences awaiting replication include Rey (2003, 2007), who measured low-temperature thermoluminescence, and Cartwright (2016), who used solvatochromic dyes. These reports are typical of these types of studies in that they involved technically complex analytical methods and lacked important control measures and other safeguards against error, for example, in neither was the experimenter ‘blinded’ and in Cartwright (2016) the control solution was not repeatedly diluted and succussed as the remedy was. The use of complex methods in the absence of control measures predisposes to false-positive findings, especially if the experiments were carried out by believers in homeopathy, and such false-positive findings may be more likely to be published by journals specifically concerned with homeopathy or other complementary and alternative therapies, if the journal editors and peer reviewers do not fully understand the methodology (Lee and others 2002, Smith 2006, Doehring and Sundrum 2016). Such factors are why independent replication is so important – not just for homeopathy-related experiments, but for any new experimental result (Ioannidis 2005a, Prasad and Cifu 2015). Further, it is notable that, for the phenomena so far reported in such studies, there is no indication of how the phenomena – if genuine – might contribute to the claimed medicinal effects of the remedies.

Montagnier and others (2009) claimed to find electromagnetic signals from bacterial deoxyribonucleic acid in extreme dilutions. However, from a physical and technical perspective, this work is suspect (Grimes 2012) and this finding has not been independently replicated.

We note that the curative property must interact with the physical world in order to: pass from the starting material into a remedy; pass from dilution to dilution; increase in potency as a result of succussion; and cure diseases in a patient. Therefore, if this curative property exists, the failure of modern science to detect any trace of it in remedies appears astonishing. Any genuine difference between ultra-dilute remedies, or between such a remedy and its diluted diluent-only control, would be a fundamentally revolutionary finding for the fields of physics and chemistry in general. And if the curative property was found to be able to treat almost any disease of a huge range of aetiologies and pathogeneses – infectious, inflammatory, toxic, neoplastic, structural, congenital – that would be a fundamentally revolutionary finding for the fields of biology and medicine. However, there is no proven, replicable difference between ultra-dilute homeopathic remedies and control solutions made from the same diluent to explain the alleged curative actions of homeopathic remedies, and the ‘potentised’ curative property in homeopathic products remains undetected by modern science.

Numerous studies claim to show that homeopathic remedies have effects on in vitro cell preparations or experimental animal models (many such studies are listed by Malik 2012, and Rational Veterinary Medicine 2017). These studies typically show the same types of design weaknesses as those searching for special physical and chemical properties of the remedies themselves, and are without independent replication to confirm the effects reported. There have been some ‘false alarms’, but none have been convincingly replicated. Possibly the best known case was a paper published in Nature (Davenas and others 1988) in which the eminent immunologist Benveniste’s research group claimed that human basophils produced histamine when exposed to anti-immunoglobulin E even at a dilution of 60c. The findings were later shown to be due to observer bias (Maddox and others 1988). The data had been generated by an unblinded technician and subsequent multiple attempts by independent researchers to replicate the findings failed (Maddox and others 1988, Hirst and others 1993). Benveniste continued to claim that the results were authentic (Kayne 2006) and, later, that he could encode the effect electronically and transmit it over telephone lines, to turn water into a homeopathic remedy remotely (Jonas and others 2006). Belon and others (2004) and Ennis (2010) concluded that ultra-dilute histamine solutions may modulate basophil activation; but the effect was small and inconsistent. It was further concluded that well-controlled, large-scale studies are required to confirm the small and inconsistent effect. These have not been forthcoming.

#### Contaminants

In the preparation of homeopathic remedies the starting ‘active’ is rarely a pure or sterile substance, and so will be contaminated with a variety of inorganic and organic chemicals and microorganisms, but homeopaths regard these as being a natural part of the ‘active’ (Kayne 2006), presumably conceived as contributing to the curative property in the remedy. Water inevitably contains many other compounds, including dissolved gases (nitrogen, oxygen, carbon dioxide and others), inorganic chemicals (sodium, chloride, calcium, phosphate), organic molecules from animal and vegetable sources, and possibly living microorganisms as sterility is not generally claimed for homeopathic products. Many homeopathic remedies are prepared using distilled water, which will eliminate or reduce some of these contaminants. In general, homeopaths do not appear to regard contaminants in the diluent as contributing to their remedies’ medicinal effects, even though any contaminants present in early dilutions would presumably be ‘potentised’ by the later dilutions and succussions. However, there is a class of contaminants that some homeopaths (Anick and Ives 2007, Bell and Koithan 2012, Bell and others 2015) have speculated may provide a mechanism for the action of remedies diluted beyond the Avogadro limit – nanoparticles either of the ‘active’ and/or of silica from the glass vials in which the remedies are diluted and succussed. Molecules of the starting substance, in the form of nanoparticles, have been found in some Indian commercial ultra-high dilution (30c and 200c) remedies made from metals (Chikramane and others 2010, Temgire and others 2016). The presence of such nanoparticles presumably constitutes either incomplete dilution or contamination by some of the starting substance after dilution. Bell and Koithan (2012) and Bell and others (2015) speculated that nanoparticle contaminants in homeopathic remedies could constitute a mechanism for transfer of information via ultra-dilute remedies from the starting substance to the patient, but have provided no evidence that it does so.

#### Selection of homeopathic remedies

In homeopathic practice, remedies are selected on the basis of patients’ symptoms and signs (signs only in animals), and on other characteristics of patients such as their temperament, preferences in life or previous experiences (Gregory 2008, Lilley 2008, Nicolai 2008, Reilly 2008, Owen 2015a, b, c, British Association of Homeopathic Veterinary Surgeons 2017). The overall ‘symptom picture’ of the patient is matched as closely as possible to the ‘symptom picture’ for the remedy, which is the collection of signs and symptoms that the remedy is believed to the able to treat, as listed in homeopathic Materia Medica (Owen 2015a, b, c). This is the practical application of the principal that ‘like-cures-like’ – that signs and symptoms can be cured by a remedy prepared from a substance that caused those signs or symptoms in healthy individuals. The ‘symptom picture’ for a remedy is primarily determined by a homeopathic ‘proving’ in which healthy volunteers take the substance or, more commonly, a remedy prepared from that substance, and then record their thoughts, feelings and signs (Hahnemann 2002, Kayne 2006, Lilley 2008, Riley 2008, Sherr 2015).

Most provings are conducted using an ultra-dilute remedy, not the undiluted starting material. Some remedies appear to have never been subject to ‘provings’, including some of the common homeopathic remedies (Campbell 2013), and toxicological observations or ‘therapeutic responses’ also contribute all or part of the ‘symptom picture’ for some remedies (Belon 1995, Kayne 2006, Campbell 2013). Importantly, the ‘symptom picture’ does not just consist of the symptoms and signs associated with the illness the patient may have, but includes other characteristics of the patient; for example, their temperament, preferences in life, or previous experiences, most of which conventional medicine would regard as incidental to the illness being treated (Gregory 2008, Lilley 2008, Nicolai 2008, Reilly 2008, Owen 2015a, b, c, British Association of Homeopathic Veterinary Surgeons 2017). For this reason, identical symptoms and signs of illness may be treated with different remedies in different subjects. The inclusion of these other factors in choice of remedy is a large part of what homeopaths mean when they refer to their therapy as being ‘holistic’; that is, treating the whole individual, but from a conventional medicine viewpoint this simply introduces a further degree of arbitrariness into the selection of remedies. From a scientific perspective, there seems to be no reason why ‘like’ should cure ‘like’ and the very concept of treating an illness with a substance (yet alone a highly-diluted remedy made from that substance) that reportedly created similar signs and symptoms in healthy volunteers appears arbitrary.

From the above considerations, it is clear that any mechanism by which homeopathic remedies act must differ fundamentally, not only from the fundamental principles of pharmacology, but also from the mechanisms of action of endogenous chemicals, such as hormones and neurotransmitters – their action cannot be based on conventional mechanisms of biochemistry and physiology, either in the patients’ body or in disease causing organisms.

### Pharmacology

When drugs are used therapeutically, they may treat either the underlying cause of disease/malfunction or the symptoms or signs (signs only for animals) of disease. The armamentarium consists of widely diverse classes of drugs, each with discrete mechanisms of action and targeting specific biochemical pathways in the body or in/on a disease-causing organism. Thus, antimicrobials, anthelmintics, anaesthetics, analgesics and hormones all work in fundamentally the same way (molecule to molecule interaction) but on differing biochemical pathways. Even within a general group, such as analgesics or antibiotics, the biochemical pathways differ for each subclass of agent. In contrast, in the homeopathic belief system, all remedies appear to be conceived of as acting via a single process that, as discussed above, is typically described in terms of balancing ‘energies’ or restoring ‘vital force’.

The properties of drugs and the science underpinning their use have been described in innumerable peer reviewed publications, the rate of which has accelerated in recent years. Counting only those drug-based papers classified as pharmacological, the numbers identified in Web of Knowledge were 167 in 1950, 44,426 in 1980 and 90,931 in 2010. Of these, the number (and percentage of the total) classified as veterinary pharmacology were 0 (0 per cent), 282 (0.635 per cent) and 3630 (3.992 per cent) (Lees and others 2013, Toutain and others 2016b).

Flower (2013) reviewed the basic principles of pharmacology; for reviews of veterinary aspects, see Anon (2004) and Cunningham and others (2010). The two pillars of pharmacology are pharmacodynamics and pharmacokinetics.

Pharmacodynamics is the science of drug action on the body or on a parasite/microorganism on or in the body; it is based on the concept that drug molecules interact with cellular molecules. Pharmacodynamics is studied qualitatively and quantitatively at submolecular, molecular, organelle, cell, organ/tissue and whole animal levels. Drugs act in the same manner as hormones, neurotransmitters and autacoids (local hormones) on receptors or enzymes, either to stimulate (an agonist) or to block (an antagonist) them or, for a few drugs, to do both (partial agonists).

The key pharmacodynamic properties of all drugs are: efficacy – this includes effectiveness, action on the receptor, ability to produce a response and the magnitude of the response attainable; potency – amount of drug required to produce an agonist or antagonist response, usually measured as EC_50_ or IC_50_ (50 per cent of maximum attainable excitatory or inhibitory responses, respectively); and sensitivity – the steepness of the relationship between concentration or dose and response. In the laboratory, the drug-response relationship is usually quantified in concentration/effect terms; in the animal it is usually monitored as dosage versus effect. For a large majority of drug/receptor interactions, the log of concentration relates to arithmetic response in a sigmoidal manner (a monotonic relationship), and there is usually a threshold concentration, below which no effects occur – even the threshold concentration typically being far higher than the concentration of the ‘active’ present in most homeopathic products – and the dose-response curve showing increasing effect with concentration; that is, the opposite of the concentration/effect relationship claimed for homeopathic products.

For some drug actions, the dose-response relationship is not S shaped; but inverted U or J shaped (Calabrese and Baldwin 2001, Calabrese 2005). Vandenberg (2014) described non-monotonic dose/response curves (NMDRCs) for natural hormones and endocrine disrupting chemicals (EDCs) in biological systems, including cultured cells, whole organ cultures, laboratory animals and human populations. She provided evidence for NMDRCs in the EDC literature, specifically for bisphenol A, and questions the current risk assessment practice where ‘safe’ low doses are predicted from high dose exposures. It has been suggested (Bellavite and others 2010, Calabrese and Jonas 2010) that this phenomenon of hormesis – a dose response phenomenon characterised by a reversal of response with concentration, resulting in either a J shaped or an inverted U shaped dose response curve such that a response may increase with dilution over a limited range at low concentrations – provides plausibility to homeopathy. However, none of the examples of hormesis go beyond or even near the Avogadro limit. Moreover, the magnitude of hormetic effects is small (Calabrese and Baldwin 2002) and hormesis occurs over a very limited range of concentrations (Calabrese and Baldwin 2002); in most cases the reversal of the effect with increasing concentration occurs over less than a 10-fold range (corresponding to the smallest unit of dilution, 1x, of homeopathic remedies) and hormesis rarely extends over two orders of magnitude (corresponding to a 1c homeopathic dilution), let alone the many orders of magnitude over which homeopathic products are alleged to become more potent with increasing dilution. In addition, the shape of the dose response curve differs from that of the monotonic negative ‘dose-response’ relationship claimed for homeopathic remedies. Finally, hormesis is a spontaneous natural phenomenon, which does not require homeopathic ‘potentisation’ in order to occur.

It could be argued that conventional vaccines are ‘homeopathic’ because they are made from something that can create, in healthy individuals, signs and symptoms of the disease the vaccines are used to prevent. However, vaccines are not like the thing they are used to prevent, they are the very thing (or a part of, or a modified version of, the thing) they aim to prevent. Vaccines work in a well-characterised, scientifically plausible way, by presenting antigens to the body’s immune system. Homeopathic remedies, including the nosodes (Kayne 2006) that are the homeopathic (strictly ‘isopathic’ as they are made from something considered to be involved in causing the illness, for example, a mosquito may be used to make a nosode to prevent malaria) alternative to vaccines, do not employ that mechanism. Conventional vaccines may contain relatively small amounts of the antigen, but it is still very much greater than the Avogadro limit, and they are not efficacious if diluted below a certain threshold. These properties make conventional vaccines entirely different to homeopathic remedies.

Pharmacokinetics is the science of drug absorption into, and fate within, the body. It encompasses dissolution, absorption, distribution and elimination processes, the latter comprising biotransformation (metabolism) and excretion pathways. Biotransformation involves principally the liver but other organs, such as the kidney and lung, may also contribute. Moreover, metabolism normally renders drugs less active or inactive but, in the case of prodrugs, biotransformation provides or enhances activity. Excretion involves, for most drugs, renal and/or hepatic pathways (ie, elimination in urine or secretion into bile, respectively). In ruminants, drug elimination in milk is significant for establishing a withholding time in relation to human consumption.

These pharmacokinetic processes have been studied extensively, both quantitatively and qualitatively. Pharmacokinetic processes, which have been defined and quantified for a wide range of drugs in a wide range of species, include clearance, absorption half-life, elimination half-life, volumes of distribution (central, area and steady state) and bioavailability (percentage of administered dose absorbed systemically). As well as inevitable intra-animal (eg, day-to-day) variation and intra-species (eg, dog to dog) differences, many profound inter-species differences (eg, dog to cat) in pharmacokinetic profiles exist. Moreover, there is increasing evidence of pharmacokinetic dependency on factors, such as age, breed, size and health status. For example, population pharmacokinetics quantifies breed differences, together with those associated with diseased compared to healthy animals. Indeed, for one drug, celecoxib, within breed differences were reported in healthy beagle dogs; fast and slow metabolisers within the single breed were identified (Paulson and others 1999). For another drug of the same class, mavacoxib, pharmacokinetic differences have been defined between small and larger breeds in the clinical population (Lees and others 2015a).

By quantifying the contribution of these factors to variability, and by linking pharmacodynamics with pharmacokinetic properties, a rational basis for designing dose schedules for use in the clinical population, is provided. This approach has been used to predict target attainment rate doses of antimicrobial drugs for a given level of bacterial kill (say 99.9 per cent) in a given percentage of the clinical population (say 50 or 90 per cent) (Lees and others 2015b).

In contrast, there appears to be no equivalent of pharmacokinetics in homeopathy; because the ‘curative property’ of homeopathic remedies is undetectable, it is not possible to measure whether it varies with location in the body, or over time, and so equivalents of parameters and variables such as bioavailability, half-life and clearance cannot be determined. For this reason, the posology of homeopathic remedies – the ‘potency’ used, frequency of administration and duration of treatment, must be entirely empirical, if not arbitrary. Kayne (2006) and Nicolai (2008) discuss homeopathic posology in human patients. Gregory (2008) observed that animals appear to need more doses of homeopathic remedies than humans, that the smaller species need much more frequent dosing, and that horses are far more sensitive to homeopathic remedies than any other species.

## Problems and pitfalls in assessing data

Irrespective of the differing proposed mechanisms of action of homeopathic and drug-based products, the means of assessing clinical efficacy and effectiveness should be applicable to both. These should include the experience of clinicians in their daily practice, as well as well-designed and statistically evaluated clinical trials incorporating appropriate animal numbers and control treatments.

The ability of clinicians to accurately assess the efficacy of therapies in practice is known to be highly unreliable, as demonstrated by the many examples of therapies thought to have been effective by the doctors that used them that were later proven ineffective or even actively harmful (Doust and Del Mar 2004, Prasad and Cifu 2016), and the high incidence of misdiagnosis – and hence mistreatment – revealed by autopsy studies (Shojania and others 2003). There are many causes of the unreliability of clinicians’ assessment of treatment efficacy, and many of them are cognitive in nature, the result of biases that are inherent to human perception and reasoning (Kahneman 2012, Matute and others 2015) and that influence clinicians’ judgements in their everyday work (Croskerry 2003, Gay 2006, Hartman 2009, McKenzie 2014, Canfield and others 2016, Saposnik and others 2016). The degree of reliability of judgment varies with the type of therapy; if a drug’s response occurs very quickly after administration, is very large, very repeatable and markedly different to the animal’s natural variation over time, it is easy for the clinician to make accurate judgments on efficacy. Thus, practitioners can judge the effectiveness of an intravenous general anaesthetic, such as alfaxalone or propofol, very clearly. Within seconds of administration, the animal’s state changes from conscious and responsive to unconscious and unresponsive, in a highly repeatable way that would not occur if the animal had not been given the anaesthetic. Assessing the response of a dog in severe acute pain to an injection of a strong analgesic, such as morphine, is also generally reliable, although the response is slower and less readily observed. However, certainty declines as the time to, size of, and repeatability of a drug’s effect decreases, and as the animals’ variation over time in the relevant characteristics increases. Thus, it can be difficult to assess the effect of, say, a nutraceutical joint supplement on a dog’s signs of arthritis six weeks after commencing dosing.

In the presence of uncertainty about treatment benefits resulting from the fact that they are superimposed on natural variation of the animals’ signs – and most illnesses will improve because of natural healing mechanisms – various psychological biases result in clinicians tending to over-estimate the effects of the treatments they have given. There are many such biases (Rudolf 1938, Pinto 2001, Gay 2006, Kahneman 2012, McKenzie 2014, Matute and others 2015, Canfield and others 2016, Saposnik and others 2016); a particularly important example is the post-hoc ergo propter hoc error, where an expected change after giving a medicine is attributed to the medicine, whether or not the change was actually induced by that medicine. It is because of the inherent uncertainties in assessing the response to treatments, and the psychological biases that can mislead physicians in judging their effectiveness, that randomised, blinded, controlled clinical trials have been developed; these can largely, although often not perfectly, remove the effect of errors in judgment. Unfortunately, many practitioners are largely unaware of the many psychological biases that influence their everyday clinical judgments – a greater awareness of such biases would improve the practice of both veterinary and human medicine, whether conventional or alternative (Croskerry 2003, 2013, Gay 2006, McKenzie 2014, Canfield and others 2016).

The design, conduct and reporting of clinical trials is also subject to a number of biases, in particular to confirmation bias, ascertainment bias, selection bias and publication bias (Easterbrook and others 1991, Stern and Simes 1997, Ioannidis 1998, 2005b, 2014, Ioannidis and others 2001, Bekelman and others 2003, Lexchin and others 2003, Chan and others 2004, Jadad and Enkin 2007, Viera and Bangdiwala 2007, McGauran and others 2010, Sargeant and others 2010, Hróbjartsson and others 2012, Kahan and others 2015, Ahn and others 2017). For clinical trials, the ideal features (rarely achieved in veterinary medicine) are: independent investigators; blinding of the person administering the product and the individuals making the response assessment as well as those analysing the data, and also the patient in human trials; a sufficient number of treated patients (often requiring a power calculation); incorporation of a positive control (alternative drug usually of the same group) and/or a negative control (usually placebo-treated); allocation of treatments to groups on a truly random basis; appropriate use of statistics; accurate and detailed reporting of the methods and results; high-quality peer review; and replication of the trial by independent investigators. Replication of clinical trials is particularly uncommon in veterinary medicine. Objective guidelines exist for assessing randomised controlled trials (RCTs) with regard to these factors (Schulz and others 2010, Higgins and Green 2011, Sargeant and O’Connor 2014). Two further essential features of the ideal clinical trial are that: the trial design, and especially the intended primary and secondary outcome measures, be published before conducting the trial (which helps to prevent inappropriate post-hoc statistical analysis); and the trial results be published regardless of the findings. Major deficiencies in clinical trials in these two regards are being addressed by the AllTrials (2014) and VetAllTrials (2015) initiatives. It has been shown that RCTs carried out by investigators with financial conflict of interest produce more positive results than trials carried out by independent investigators (Bekelman and others 2003, Lexchin and others 2003, Ahn and others 2017). All of these considerations apply equally to the assessment of clinical efficacy or effectiveness of drug and homeopathic products.

These ideals are far from always achieved in human RCTs of conventional medicines (Ioannidis 2005b, 2014, Prasad and Cifu 2015), much less frequently in RCTs of homeopathy, and rarely in veterinary RCTs. Di Girolamo and Meursinge Reynders (2016) reviewed the effectiveness-of-intervention studies in five leading veterinary journals and five leading medical journals for the year 2013. Median numbers were 26 and 465, respectively, the veterinary studies were smaller and only 2 per cent of veterinary RCTs v 77 per cent of human RCTs reported power calculations, primary outcomes, random sequence generation, allocation concealment and estimation methods. One reason for these differences is cost; pharmaceutical companies must necessarily make a profit and these are generally much smaller on veterinary than on human medicines. Another factor is animal welfare. Can we, the best scientific approaches notwithstanding, ethically justify a placebo-controlled trial in calves with acute pneumonia or dogs with severe osteoarthritis? These issues are not easily addressed.

Trials with negative results (however useful they might be) are less likely to be published than those with positive findings, not least because of journal editors’ interest in preserving or enhancing their impact factors (Easterbrook and others 1991, Stern and Simes 1997, Ioannidis 1998, Smith 2006). Independent replication of clinical trials is important in establishing efficacy (Ioannidis 2005a, 2014, Anon 2013, Prasad and Cifu 2015). It permits a check on whether the initial study might have had false-positive or false-negative results, or shown an unrepeatable effect size. Ioannidis (2005a) reported on 49 human clinical studies. Thirty-four reported a significant positive effect, but when later retested the results were negative in seven cases and the effect sizes smaller than in the initial report in seven more. The reasons might include improved study design in the replication studies reducing false-positive findings of the initial studies. Random ‘noise’ – chance variation – will result in false positives one occasion in 20 at the P=0.05 level of statistical significance. If a study measures 10 variables, chance alone will give a 50 per cent probability that one of them will be ‘statistically significant’ unless statistical techniques are adopted to adjust for that fact – which is not always done. However, most false positives are likely due to other factors, particularly failure to fully control for confounding factors – as mentioned above – such as biases and the natural course of the diseases.

For the above reasons, doctors and veterinarians should always be vigilant and constructively critical in making assessments both on the basis of their everyday clinical experience and of clinical trial findings. The assessment difficulties are likely to be greater when the end-point measurements of efficacy are nebulous and/or subjective rather than clear and/or objective, and so the risk of erroneously ascribing a specific treatment effect to an actually ineffective medicine will be higher.

Homeopathy is most frequently used to treat chronic conditions with fluctuating signs, or acute, self-limiting conditions (Jacobs and others 1998, Mathie and others 2007, 2010). These are precisely those conditions for which assessment of treatment responses is most difficult and prone to error because of the natural history of the disease and subjective biases, and so it is particularly important that responses to therapy are not based purely on subjective assessments and anecdotal experiences of veterinarians (Mathie 2007, 2010), or on the results of poorly designed and conducted clinical trials, but rather on the results of well-designed and conducted RCTs. This is well illustrated by the example of homeopathic treatment of feline hyperthyroidism. Two prospective ‘outcome studies’ (Mathie and others 2007, 2010) – uncontrolled reports of how well practitioners and or clients believed hyperthyroid cats responded to treatment – and one case series of four hyperthyroid cats (Chapman 2011), each suggested that homeopathy is an effective treatment for hyperthyroidism. However, a well-designed, double-blinded RCT showed that individualised homeopathy had no effect on hyperthyroidism, as assessed by blood thyroid hormone level, heart rate and weight after 21 days, whereas standard methimazole treatment was effective (Bodey and others 2017).

## Peer-reviewed clinical trials and systematic reviews

As discussed above, for clinical trials in people and animals, there exist widely accepted (but not always applied) standards, procedures and guidelines on study design and conduct and the statistical evaluation of data generated, with recommended features including randomisation, blinding, positive and/or negative (placebo) controls and sufficient number of animals. These general principles are explicated in detail in various published guidelines for designing and/or assessing RCTs (for example, Schulz and others 2010, Higgins and Green 2011, Sargeant and O’Connor 2014). Systematic reviews use this type of objective methodology to formally assess the design, conduct and reporting of published controlled clinical trials to minimise the effects of bias, and there are formal, objective protocols and guidelines for conducting systematic reviews and meta-analyses (see Higgins and Green 2011, Zoonoses and Public Health 2014, PRISMA 2017). Clinical trials in both human and veterinary medicine, which have been objectively evaluated as meeting high standards and thus ensuring high-quality evidence, provide a huge body of evidence, which inevitably is not universally complimentary to drug-based products and extremely rarely supports a positive outcome from homeopathic trials.

A means of boosting animal/patient numbers is to take a number of trials of sufficient quality of design and conduct, and analyse the composite of those trials – a meta-analysis. There are several objective methods for assessing the quality of meta-analyses and systematic reviews. This is one major function of the Cochrane Collaboration (www.cochrane.org/), an international not-for-profit organisation of collaborating medical professionals tasked with determining the effectiveness of treatments, which produces systematic summaries of research literature in healthcare.

For homeopathic products used in people, there is a large base of peer-reviewed published clinical trials, and several reviews thereof (Linde and others 1997, Cucherat and others 2000, Jonas and others 2003, Shang and others 2005, Milazzo and others 2006, Ernst 2010, Mathie and others 2014, 2017). Shang and others’ (2005) meta-analysis assessed every clinical trial conducted in people published up to that time investigating the efficacy of homeopathy. Poor-quality trials were excluded to provide a demanding but fair test. Shang and others (2005) found a small positive effect of homeopathic treatments over placebo, much smaller than the positive effect of conventional treatments over placebo. Given the difficulty of completely removing bias in clinical trials, and the fact that even the best-quality trials were not ideal, their finding was consistent with residual bias affecting the trial results and the authors, therefore, concluded that the apparent benefits of homeopathy were compatible with placebo effects. However, the data reported by Shang and others (2005), in and of itself, does not allow the conclusion to be drawn that the small positive effect reported was not a specific effect of homeopathic products.

As pointed out by Hektoen (2005) ‘animal studies may…. be more useful than human studies in determining whether homeopathic remedies have specific effects in comparison with a placebo’. Mathie and others (2012) collated RCTs of veterinary homeopathy, and identified 38 substantive peer reviewed articles suitable for future review. Mathie and Clausen (2014) carried out the first systematic review of RCTs of veterinary homeopathy compared with placebo (18 RCTs, 12 therapy and six prophylaxis) quantifying effect size. Only one trial was free of vested interest (eight were unclear) and risk of bias was high in 11, low in one and unclear in six. They concluded; ‘mixed findings from the only two placebo-controlled RCTs that had suitably reliable evidence precluded generalisable conclusions about the efficacy of any particular homeopathic medicine or the impact of individualised homeopathic intervention on any given medical condition in animals’. Mathie and others (2014) also carried out a systematic review and meta-analysis of randomised placebo-controlled trials of individualised homeopathic treatments in humans. The conclusion was that they ‘may have small, specific treatment effects … the low or unclear overall quality of the evidence prompts caution in interpreting the findings. New high quality RCT research is necessary to enable more decisive interpretation’.

Thus, on the basis of evidence from RCTs, meta-analyses and systematic reviews alone, the small positive effects reported in people and animals could be the result either of specific effects of homeopathy or residual bias not fully controlled for in the trials (Cucherat and others 2000, Shang and others 2005, Mathie and Clausen 2014, 2015a, Mathie and others 2014, 2017). In light of the considerations discussed in this review and part 1 (Lees and others 2017), on: the potential for the natural history of diseases, placebo effects and subjective biases to yield artifactual positive results; the difficulties in assessing evidence and, particularly, of performing RCTs to ideal standards; and the implausibility on theoretical grounds of homeopathic remedies having any specific effect, it is overwhelmingly likely that small effects observed in the RCTs and systematic reviews are the result of residual bias in the trials. In contrast, the clinical effects claimed in veterinary practice by homeopaths are often large (Mathie and others 2007, 2010).

Mathie and Clausen (2015b) conducted another systematic review of RCTs of veterinary homeopathy, in which the control group received an intervention (active controls) rather than a placebo. They used Cochrane methods to assess risk of bias and derive effect size in 14 treatment and six prophylaxis studies. They concluded that, due to the poor reliability of the data – no trial had sufficiently low risk of bias to be judged reliable – the trials did ‘not provide useful insight into the effectiveness of homeopathy in animals’.

Doehring and Sundrum (2016) performed a review of trials of homeopathy used for the treatment of infectious diseases or growth promotion in farm animals. Of 48 studies meeting their inclusion criteria, 15 were doctoral theses and 33 were published in peer-reviewed journals, of which 18 were in journals dedicated to homeopathy or alternative medicine and 15 in veterinary journals. Their literature review specifically included a wide range of trial designs, including RCTs – eight of which had been excluded from Mathie and Clausen’s (2014) systematic review of veterinary homeopathy RCTs for not constituting reliable evidence, and lower-quality controlled studies that were unblinded and/or unrandomised and/or with a control group that was not placebo-treated, and some observational studies that had no control group. For these reasons, there was substantial potential for non-specific effects including bias, and many of the trials with findings positive for homeopathy cannot be taken as good-quality evidence that homeopathy is effective. Doehring and Sundrum (2016) found that the trials better designed to reduce non-specific effects produced results less positive for homeopathy. They also found that trials published in journals devoted to homeopathy or alternative medicine were much more likely to be positive for homeopathy than trials published in journals with a broader focus on veterinary medicine (15 of 18 trials v six of 18 trials), indicating publication bias. The trials that produced results positive for homeopathy included a very heterogenous range of diseases, remedies and circumstances, but not one of them had been replicated. Doehring and Sundrum (2016) concluded there was insufficient evidence to recommend that homeopathy be used to replace or reduce antibiotics in the treatment of farm livestock.

## Ethical and negative aspects of pharmacology and homeopathy

As discussed by Jacobs and others (1998), homeopathy in people is used most frequently in chronic and acute, self-limiting conditions. Likewise, in small animal practice, there is a high prevalence of chronic diseases, including allergies and joint diseases, for which drug-based therapeutics offers real but often only palliative care. This can stimulate pet owners to search for and even insist on alternative medical treatments (Hektoen and others 2004, Hektoen 2005). In farm animal medicine, homeopathy has found favour with some organic farmers, who rightly perceive the downsides of conventional therapeutics, while being reluctant to acknowledge the upsides. The disadvantages of drug-based therapeutics are, in some cases: failure to achieve ‘cure’ (ie, less than 100 per cent efficacy); toxicity to the treated animal; trace amounts of drugs and their metabolites in meat and milk; and emergence and spread of antimicrobial and anthelmintic resistance, not only compromising the success of animal therapy but involving spread of resistance factors into the environment (Toutain and others 2016a).

Hovi and Roderick (1999) reported that homeopathy was the main alternative to antibiotic therapy on UK organic farms, accounting for 50 per cent of mastitis treatments. The use of homeopathic products may be ideologically based (a preference for ‘natural’ products or a dislike of drugs as ‘chemicals’), a result of the above mentioned disadvantages of conventional therapies, and/or economically based, using inexpensive homeopathic products and also no requirement to adhere to milk and meat withholding periods.

The vast majority of medical scientists, doctors and clinical veterinarians support the judicious use of drug-based products and vaccines as the mainstay of veterinary therapeutics. However, cultural and social differences occur between countries, and complementary therapies, including homeopathy, are more extensively accepted and practised in, for example, France, Italy, Germany and India than in the UK.

### Pharmacology

Despite all the welfare benefits of safe anaesthesia, control of pain, effective prevention and cure of diseases caused by microorganisms, helminths and ectoparasites and many other benefits, there are significant downsides to the use of drug-based veterinary products. There will be many occasions when the drugs themselves are ineffective or effective suboptimally. Many drugs are being used by doctors and veterinarians despite an insufficient evidence base to prove their efficacy, some of which will go on to be proven ineffective (Prasad and Cifu 2015). There are side effects for virtually all drugs, which may be life threatening. Side effects may be idiosyncratic (rare but marked toxicity with clinically recommended dosage) but more usually are dose-related. Side effects of conventional medicines arise from biochemical and physiological mechanisms, and many drugs have characterised toxicological thresholds and dose/response relationships in the same way as they have pharmacological thresholds and dose/response relationships.

A negative aspect of current global concern is the emergence of resistance to antimicrobial drugs. Relative to people, this is less of a concern in terms of effective treatment of microbial disease in animals, for which many drugs have retained a high level of efficacy, but a major concern is the impact on the environmental resistome, through the extensive use of antimicrobial drugs, in particular in farm animal medicine. The significance of this as a potentially major public health issue is increasingly recognised (Toutain and others 2016a). For therapies of all classes, there is the universal dimension of clients’ expectations that they always should be administered tablets or an injection when they visit the veterinarian or doctor. Carefully managing this expectation would reduce the unnecessary dispensing of drugs, most important for antimicrobial drugs, thereby reducing the global problem of antimicrobial resistance.

### Homeopathy

Homeopaths argue that, at least homeopathy does no harm. This is questionable. Although it is unlikely that most homeopathic remedies contain substances that could have a specific toxic effect. The World Health Organization (2009) advises ‘there are a few aspects of the production of homeopathic medicines that could constitute potential safety hazards. Firstly, not all homeopathic medicines are administered at a high dilution. Sometimes, a homeopathic medicine made from source material, such as a mother tincture, is administered in the most concentrated form… Secondly, homeopathic medicines are made from a wide range of natural or synthetic sources including fungi, bacteria, viruses and plant parasites… Some of these source materials constitute potential safety hazards, even at high dilutions’.

In human patients, placebo effects can be of genuine value, as discussed in part 1 of this review (Lees and others 2017). However, in veterinary medicine it will be very rare – unless specifically organised by prior conditioning of the animal – that circumstances will be such that a genuine placebo effect can be of benefit. In human medicine also, there can be a counselling/psychotherapeutic aspect to homeopathic consults that can be of benefit to the patient, and in veterinary medicine such consults can be of benefit to animal owners, but not directly to the animals. Indeed, placebo effects engendered in owners – known as ‘caregiver placebo effects’ (Conzemius and Evans 2012, Gruen and others 2014, 2017) – can actually be detrimental to their animals because the owners perceive an improvement that may not be present. Probably the most harmful aspects of homeopathy are the delay in treatment, or the withholding of conventional treatments completely, when ineffective homeopathic remedies are given to animals that may be suffering, in place of effective conventional treatments, as established by scientifically demanding regulatory requirements and/or published clinical trials. Similarly, use of an ineffective homeopathic preparation, in place of effective conventional vaccination, and withholding other prophylactic treatments such as wormers, may be harmful to animal welfare. Use of an ineffective treatment in these circumstances is unethical, particularly because animals, like young children, have no voice in the treatment they receive. Moreover, clients, including sometimes desperate owners, should not be offered false hope through ineffective products. It is most unlikely that a veterinarian prescribing a homeopathic product will inform the client that it is lacking in specific efficacy. For clients who insist on homeopathic treatments, even if fully informed, in veterinary medicine, it is questionable whether client demand should take precedence in those cases where there are clear animal welfare issues.

Homeopaths commonly recommend that drug-based products should actively be avoided. The Academy of Veterinary Homeopathy Standards of Practice (2017) states, ‘Concurrent treatment with many drugs, herbs, acupuncture and other types of intervention can reduce the effectiveness of homeopathic medicines … only those medicines that are homeopathic to the patient’s condition should be administered … Concurrent drugs, herbs, and electromagnetic applications should be avoided, when possible, to prevent the possibility of interfering effects on the life force …’ It is common for veterinary homeopaths to claim that vaccination is harmful and that commonly used veterinary medicines interfere with homeopathic treatment (for example, Gregory 2008, 2013b); ‘it is also well known among homeopaths that the action of homeopathic remedies is severely reduced by concurrent administration of NSAIDs or indeed any other anti-inflammatory agents, such as corticosteroids or ciclosporin’ (Gregory 2013b).

Another negative aspect of homeopathy is that, when offered by veterinarians, it devalues conventional veterinary qualifications through the use of ineffective and irrational treatments – failing to differentiate veterinary surgeons from unlicensed healers and so undermining confidence in mainstream medicine (Chambers 2013). In veterinary medicine, homeopathy is practised by a small minority of practitioners, with postnominals granted by homeopathic organisations, but used alongside recognised veterinary qualifications, without any distinction being made between the qualifications that are recognised by veterinary regulators and those that are not.

For discussion of the ethics of the practice of homeopathy on human patients, see Shaw (2010) and Smith (2012). Among other problems, both argue that the practice of homeopathy by doctors is a waste of medical resources and that, when doctors practice homeopathy but fail to acknowledge the placebo effect as the principal basis for efficacy, they are being economical with the truth, providing homeopathy with unwarranted credence, and weakening support for science-based and evidence-based medicine. These factors all apply to veterinary practice as well. However, in human medicine there are, at least, recognised placebo effects, and the counselling/psychotherapy aspects of homeopathic consultations, that may be of value to those patients who seek out homeopathy. In contrast, in veterinary medicine, these effects are of no benefit to animals, as veterinary homeopaths are effectively treating owners, not animals, when prescribing ineffective remedies for the owner’s animals.

## Acceptance of homeopathy

The doctor and science writer Goldacre (2008) wrote, in his book Bad Science, ‘homeopathy is perhaps the paradigmatic example of an alternative therapy: it claims the authority of a rich historical heritage, but its history is routinely rewritten for the PR needs of a contemporary market; it has an elaborate and sciencey-sounding framework for how it works, without scientific evidence to demonstrate its veracity; and its proponents are quite clear that the pills will make you better, when in fact they have been thoroughly researched, with innumerable trials, and have been found to perform no better than placebo’.

The practice of homeopathy confronts us with two clear, mutually exclusive hypotheses. One is that homeopathic remedies are genuinely effective. However, that hypothesis is extremely implausible, for all the reasons discussed in this two-part review. The other hypothesis is that homeopathy has no effect beyond placebo effects and that homeopaths’ judgement of the efficacy of their remedies is incorrect. This is a simple and highly plausible hypothesis, for all the reasons discussed in this two-part review, which appears consistent with all available evidence.

Open discussion, debate and criticism of all medical treatments must be encouraged. Opinions based on anecdote and experience are unreliable. Conclusions on efficacy and safety will have most value when they are based on sound science and objective weighing of all available evidence. Science is bottom up and ‘evolutionary’, building upon previously established facts using the ‘parsimony principle’ – the simplest explanation possible. Homeopathy, on the other hand, is top down and faith-based; governed by arbitrary laws, invented by the founder, Hahnemann, which are immutable. As such, homeopathy is not just unscientific, it is a genuinely mystical belief system.

There are clear differences between the laws of homeopathy and the scientifically determined laws of nature. Laws of nature are not arbitrary; they are based on formal observation of phenomena, have been thoroughly tested and for most of them the underlying mechanisms have been elucidated. No law of nature is inconsistent with physics, chemistry and biology, and many are related to each other in ways that show them to be part of the same overall natural system. In contrast, the three laws governing homeopathic remedies (‘like-cures-like’, dilution/infinitessimals and succussion) are arbitrary. They have not been subjected to rigorous testing, there is no known underlying mechanism(s), and the Law of Infinitessimals in particular is not only arbitrary, but explicitly contrary to the scientific understanding of physics, chemistry and biology. Furthermore, the three laws of homeopathy have no apparent relationship to each other. Thus, there appears to be no a priori reason why a curative property that would be efficacious on the basis of the particular type of ‘like-cures-like’ favoured by homeopathy should also have stronger effects when highly diluted and/or require succussion for its healing effect to be activated. And no a priori reason why potentisation of that curative property requires both dilution and succussion.

No theory to explain the alleged specific healing effects of homeopathic remedies is compatible, even marginally, with what is known of bodily functions or the properties of disease-causing organisms. The unknown ‘curative property’ of homeopathic remedies is supernatural in that it acts ‘beyond scientific understanding or the laws of nature’ (Oxford Dictionaries 2017). Its supernatural properties include: it is present throughout most if not all of the physical world but is undetectable by science even though it must interact with physical matter to have the properties attributed to it by homeopaths; it increases in potency with increasing dilution; and it can be manipulated by the initiated – trained homeopaths – in order to treat almost any of a huge variety of diseases of widely differing aetiologies and pathogeneses, without doing harm.

‘Magic’ is commonly defined as ‘the power of apparently influencing events by using mysterious or supernatural forces’ (Oxford Dictionaries 2017). In anthropology – the academic study of aspects of humans within past and present societies, which field includes magical and religious beliefs – ‘magic’ generally refers to ‘beliefs and behaviours in which the relationship between an act and its effect is not empirically or scientifically verified but, from a Western perspective, rests on analogy or a mystical connection’ (Moro 2012). Thus, ‘like-cures-like’ – in the absence of a scientific explanation and resting entirely on analogy – is an explicitly magical belief in the ancient tradition of sympathetic magic (Fraser 1922).

The practice of homeopathy by veterinary surgeons is accepted by veterinary regulatory bodies around the world, including the Royal College of Veterinary Surgeons in the UK (Viner 2016). The issues discussed in this article and its companion (Lees and others 2017) raise two key questions. First, is it appropriate for veterinary professionals to treat animals on the basis of mystical beliefs requiring invocation of supernatural forces. It can be argued that doing so diminishes our science-based profession as a whole. As expressed by Hektoen (2005), ‘it is important for the veterinary profession to discuss the question of whether veterinarians, as medical professionals, should recommend or practise a theory with no scientific basis, and to what extent clients’ preferences and motivation for treatment should be acknowledged’. Likewise, the Connecticut Veterinary Medical Association (2013) advised that ‘the veterinary profession has an obligation to society and to our clients to acknowledge the conclusions of science even when there is not absolute unanimity within the profession. If we wish to retain the trust of the public, upon which our work depends, we must demonstrate that our recommendations are based on sound science and that we are willing to put the welfare of our patients and clients first even when some of our colleagues object’.

Second, if homeopathic remedies have no specific effect; and it is rare that placebo effects exerted through the owner will be beneficial to the animal and, more commonly, the placebo effects on the owner will be irrelevant or even harmful to the animal; and use of homeopathic remedies may delay or prevent use of proven-effective conventional treatments in ill animals, is use of homeopathy by veterinary surgeons acceptable? If it is, the principle of informed consent implies that the prescribing veterinary surgeon should inform clients that homeopathic products have no benefit beyond non-specific effects and to fully inform clients of the nature of placebo effects and that they will typically have no effect on their animal(s) (Whiting 2012). It would, moreover, be ethical to insist on an immediate recourse to a proven conventional therapy when any form of pain or other suffering is diagnosed. It is not clear if this manner of proceeding is generally observed by homeopathic veterinary surgeons at present, and it cannot be doubted that the use of ineffective practices by veterinary surgeons, in the sincere belief that they are effective, is capable of compromising animal welfare.

## Conclusions

Homeopathy appears to be one of many examples from the history of medicine, of therapies, conventional and otherwise, which were thought to be effective but were later proved to be ineffective or even harmful. One doctor, Samuel Hahnemann, working more than 200 years ago, at a time preceding modern science and medicine, proposed a vitalist system of therapy that has persisted to the modern day despite being incompatible with the modern scientific understanding of the world, and despite the failure of high-quality clinical trials to demonstrate efficacy for even one medical condition (House of Commons Science and Technology Committee 2010, Australian Government 2015; see supplemental material for this article). The homeopathic curative property is not detectable by scientific methods and, although homeopaths report that their remedies are effective when used in their practice, efficacy beyond placebo is not apparent in well-controlled clinical trials, which eliminate biases and other non-specific effects. In human medicine, there may be a place for the counselling/psychotherapeutic aspects of homeopathic consults and the placebo effects generated by homeopathic products in patients who believe in such treatments, but in veterinary medicine these factors are unlikely to benefit patients, and the use of homeopathic products in veterinary medicine is contrary to best evidence, irrational, and inconsistent with current scientific and medical knowledge (Chambers 2016, Whitehead and others 2016).

The pharmacological basis of therapeutics is, in virtually every respect, the opposite of homeopathy. In the great majority of cases it is based on increased effect provided by increased dose or concentration up to a ceiling, the maximum attainable response. Doses are determined by the application of data on each drug’s pharmacodynamic and pharmacokinetic properties, established on a species basis. Additionally, increasingly recognised is the need sometimes to adapt dose not only for bodyweight but also for disease severity, condition of animal, as well as age and breed differences in pharmacodynamics and pharmacokinetics. Drug-based therapeutics emerged by evolutionary processes from Materia Medica, which it has supplanted, and it will continue to evolve with advances in clinical and non-clinical sciences. As reviewed in this article, there are many disadvantages to the use of drug-based products in veterinary medicine. However, their benefits and their side effects are based on principles compatible with modern scientific knowledge. They are subject to rigorous evaluation for quality, safety and efficacy by regulatory authorities (unlike homeopathic remedies; see supplemental material for this article). They have contributed greatly to animal welfare and the relief of suffering.

10.1136/vr.104279.supp1 Supplementary material Appendix 1: UK Licensing requirements for drugs and homeopathic remedies. Appendix 2: Assessments of homeopathy by governmental, regulatory and veterinary professional bodies. To view please visit the journal online http://veterinaryrecord.bmj.com/content/181/7/198

## Conflict of interest statement

None of the authors of the article has a financial or personal relationship with other people or organisations that could inappropriately influence or bias the content of the paper. D. Chambers and M. Whitehead are members of the Campaign for Rational Veterinary Medicine.
