# Supplementary material for: Comparison of veterinary drugs and veterinary homeopathy: part 2
Source: Vet Rec. 2017 Aug 19;181(8):198–207. doi: 10.1136/vr.104279 (PMC5738588; doi:10.1136/vr.104279)
Supplement: Supplementary material — Appendix 1: UK Licensing requirements for drugs and homeopathic remedies. Appendix 2: Assessments of homeopathy by governmental, regulatory and veterinary professional bodies. To view please visit the journal online http://veterinaryrecord.bmj.com/content/181/7/198 [file vetrecj104279supp001.pdf]

Supplementary material for Lees and others (2017) Comparison of veterinary drugs and veterinary homeopathy: part 2. Veterinary Record doi: 10.1136/vr.

Appendix 1: UK Licensing requirements for drugs and homeopathic remedies

As well as establishing efficacy, both in pre-clinical studies and in clinical trials in each target species, the pharmacodynamic and pharmacokinetic approaches outlined in the main body of this review (Lees and others 2017) are required by regulatory bodies as the basis for selecting the dose of drug-based remedies. In addition, extensive studies are required pre-licensing to establish: (1) the quality of licensed veterinary products (purity, stability, shelf life etc.); (2) acceptable safety of drug products at clinical dose rates; and (3) drug and metabolite depletion profiles from edible tissues of food producing species, as required to establish meat and milk withholding periods. To this can be added the need for many products (notably but not exclusively those used in farm animal medicine on a group treatment basis) to have demonstrably minimal impact on the environment, in respect of effect on and persistence in the soil, waterways and even the air we breathe - the environmental resistome (Toutain and others 2016). Additionally, some drugs and their metabolites are toxic to fish and wildlife.

The stringent and costly but necessary licensing procedures/requirements are overseen by regulatory bodies; the Food and Drug Administration/Committee for Veterinary Medicines (FDA/CVM) in the USA, the European Medicines Agency/Committee for Veterinary Medicinal Products (EMA/CVMP) in Europe, and similar bodies in other countries at the national level, for example the Veterinary Medicines Directorate (VMD) in the UK. Each adopts high standards for establishing safety, quality and efficacy. These bodies issue many necessary guidelines on all aspects of licensing, as discussed by Jukes (2011).

In the UK, the authorisation of veterinary medicines is the responsibility of the VMD. On the VMD's Product Search page (Veterinary Medicines Directorate 2017), under a search by 'Therapeutic Group', the therapeutic group listed for homeopathic products is "N/A (Homeopathic authorisation)". The VMD lists on its website seven homeopathic products all in the General Sales List (GSL) category; each can be used at any dilution. However, there are many hundreds more homeopathic remedies with 'grandfather rights' (these were marketed before the Veterinary Medicines Regulations were introduced in 1994)

and the VMD maintains a list of them. The VMD seeks to ensure that medicinal products are safe and that unsubstantiated claims for efficacy are not allowed. The Veterinary Medicines Regulations 2013 state: "***Homeopathic remedies :***

53.—(1) A homeopathic remedy registered under these Regulations must be labelled in accordance with this paragraph.

(2) There must be no specific therapeutic indication on the labelling or in any information relating to it.

(3) The labelling (or labelling and package leaflet) must contain the following…

(a) the words 'homeopathic remedy without approved therapeutic indications for veterinary use'…

The latest Guidance on the Homeopathic Registration Scheme (Veterinary Medicines Directorate 2015) states "***A single registration may cover multiple dosage forms and routes of administration and different degrees of dilution providing they are all derived from the same homeopathic stock or stocks. To be eligible for registration a veterinary homeopathic remedy (VHR) must be:***

- ***prepared from substances called homeopathic stocks using a homeopathic manufacturing procedures described in an official European Pharmacopoeia***
- ***administered by a route described in such a pharmacopoeia, such as an oral or topical form***
- ***sufficiently dilute to guarantee safety, i.e. may not contain more than 1 part in 10,000 of the mother tincture.***

Remedies classified as sarcodes or nosodes do not need to be registered". (Sarcodes are remedies prepared from healthy animal tissues and secretions. Nosodes, which are often promoted as alternatives to vaccinations, are remedies prepared from material that causes diseases – e.g., microbe cultures, viruses, fungi, parasites – or from pathological secretions and excretions or blood or tissue from a patient with the illness to be treated or prevented.)

REFERENCES

JUKES, H. (2011) Veterinary medicines: demonstrating safety and efficacy. ***Veterinary Record*** **169**, 544-546

LEES, P., CHAMBERS, D., PELLIGAND, L., TOUTAIN, P.-L., WHITING, M. & WHITEHEAD, M. L. (2017) Comparison of veterinary drugs and veterinary homeopathy: part 2. ***Veterinary Record*** doi: 10.1136/vr.

TOUTAIN, P. L., FERRAN, A. A., BOUSQUET-MELOU, A., PELLIGAND, L. & LEES, P. (2016) Veterinary Medicine Needs New Green Antimicrobial Drugs. ***Frontiers in microbiology* 7***, 1196*

VETERINARY MEDICINES DIRECTORATE (2015) Guidance on applying to register, renew, or vary a Veterinary Homeopathic Remedy. https://www.gov.uk/guidance/apply-to-register-a-veterinary-homeopathic-remedy. Accessed December 11, 2016

VETERINARY MEDICINES DIRECTORATE (2017) Product Search http://www.vmd.defra.gov.uk/ProductInformationDatabase/Search.aspx. Accessed August 11, 2017

Appendix 2: Assessments of homeopathy by governmental, regulatory and veterinary professional bodies

Scientifically based assessments of the efficacy of homeopathic products by governmental and regulatory bodies have concluded that homeopathy is implausible in conventional scientific terms and any effect perceived or measured no greater than that of placebo or other non-specific effects. The UK House of Commons Science and Technology Committee (House of Commons 2010) concluded that "**the principle of like-cures-like is theoretically weak … this is the settled view of medical science … we consider the notion that ultra-dilutions can maintain an imprint of substances previously dissolved in them to be scientifically implausible … the systematic reviews and meta-analyses conclusively demonstrate that homeopathic products perform no better than placebos … Homeopathy should not be funded by the NHS and the MHRA should stop licensing homeopathic products**". In the UK the National Institute of Health and Care Excellence (NICE) advises the National Health Service (NHS) on medical treatments; NICE does not recommend the use of homeopathic products for the treatment of any condition. The UK NHS states (NHS England) "**There has been extensive investigation of the effectiveness of homeopathy. There is no good-quality evidence that homeopathy is effective as a treatment for any health condition.**"

UK NHS funding of homeopathic treatment – and the availability of homeopathic treatment within the NHS – has greatly declined over recent decades. Nonetheless, the NHS continues to fund a very small amount of homeopathic treatment despite the advice from NICE and the House of Commons Science and Technology Committee. At the time of writing (August 2017) a consultation is on-going regarding guidance for NHS Clinical Commissioning Groups (CCGs) on 'items which should not routinely be prescribed in primary care'. This includes homeopathic remedies on the grounds that they fall in the category of 'products of low clinical effectiveness, where there is a lack of robust evidence of clinical effectiveness or there are significant safety concerns' (NHS England 2017). If approved, this guidance to CCGs should end the already small (2016 annual spend £92,412 [NHS Digital 2017]) NHS funding of homeopathic prescriptions provided by primary care doctors in England.

As noted in Appendix 1, the Veterinary Medicines Regulations require that for homeopathic remedies registered under these Regulations "**There must be no specific**

therapeutic indication on the labelling or in any information relating to it" and "The labelling (or labelling and package leaflet) must contain the words 'homeopathic remedy without approved therapeutic indications for veterinary use'…".

In the USA, The Federal Trade Commission (2016) issued an Enforcement Policy Statement decreeing that over-the-counter homeopathic remedies must carry warnings on their packaging, stating "***that there is no scientific evidence that the product works and that the product's claims are based only on theories from the 1700s that are not accepted by most modern medical experts***". Each product will be required to carry details of any scientific proof for claimed effects. If the makers cannot back-up their claim, they will need to warn consumers there is no scientific evidence that they work.

The Australian National Health and Medical Research Council Report on evidence of the effectiveness of homeopathy for treating health conditions (Australian Government 2015) concluded: "***there are no health conditions for which there is reliable evidence that homeopathy is effective. Homeopathy should not be used to treat health conditions that are chronic, serious, or could become serious. People who choose homeopathy may put their health at risk if they reject or delay treatments for which there is good evidence for safety and effectiveness***".

The position of the British Veterinary Association (BVA) on homeopathy is succinct; "***the BVA cannot endorse the use of homeopathic medicines, or indeed any medicine making therapeutic claims, with no proven efficacy. As with any medicine, BVA believes that veterinary medicinal products must be evidence-based, with any medicinal claims made by a manufacturer supported***".

In the USA, the Connecticut Veterinary Medical Association (CVMA) proposed a resolution to The American Veterinary Medical Association (AVMA) (Connecticut Veterinary Medical Association 2013) stating "***homeopathy has been identified as an ineffective practice and its use is discouraged***". The resolution was supported by the following text (CVMA 2013): "***while habit, tradition, uncontrolled clinical experience, and anecdotes may appear to support the value of a given therapy, these sources of information are deeply unreliable. They inevitably conform to our pre-existing biases, and they can be found to support every possible practice, thus never allowing us to reject any therapy as ineffective. The dramatic and undeniable success of scientific medicine has come from relying on scientific evidence to compensate for our biases and identify which practices are effective and which are not … while homeopathy has been studied and used for 200 years, and continues to be supported by small minorities within the scientific and healthcare communities … in controlled scientific investigations it has***

failed to demonstrate effectiveness beyond placebo for any indication. This practice has failed to be validated scientifically at all levels of evidence ... a dramatically new understanding of physics, chemistry, and biomedical science would be necessary for these proposed mechanisms to be valid ... Despite the continued popularity of homeopathy among a small, but passionate community of advocates, who respond aggressively to any criticism of the practice, a growing number of veterinary, human healthcare, and governmental organizations are acknowledging that existing scientific evidence strongly supports the conclusion that homeopathy has no effect beyond placebo. Because it is unethical to offer ineffective therapies to clients, and dangerous to substitute a placebo therapy for truly effective medicine, there is a movement towards publically acknowledging that there is no reason to believe homeopathic treatment has any real value in preventing or treating disease".

Upon consideration of the CVMA's resolution and the responses of various stakeholders to it, the AVMA's Council on Research found "***there is no clinical evidence to support the use of homeopathic remedies for treatment or prevention of diseases in domestic animals***". Despite this conclusion, the AVMA rejected the CVMA's resolution on political grounds.

In the UK, section 19 of the Veterinary Surgeons Act 1966 (http://www.legislation.gov.uk/ukpga/1966/36) requires that only registered veterinarians may administer or supply homeopathy to animals (owners may administer homeopathic remedies to their animals) and, therefore, regulation of veterinary homeopaths is in the remit of the RCVS. The RCVS' position on alternative and complementary therapies (Royal College of Veterinary Surgeons 2016) emphasises "***the importance of evidence-based veterinary medicine***" and advocates a "***cautious approach***" to homeopathy, which should be used alongside, rather than as an alternative to, conventional treatment, indicating that the RCVS has concerns regarding the use of homeopathy by veterinary surgeons. The RCVS notes that there is a "***demand from some clients***" for homeopathy and that it is "***currently accepted by society***" and so finds it "***difficult to envisage any justification for banning a small number of veterinary surgeons from practicing homeopathy***". However, given that homeopathy is a vitalist, mystical belief system, the practice of which involves the use of pseudoscientific ritual to invoke a supernatural force, and given the weight of evidence indicating that homeopathy is ineffective, it is difficult to understand why homeopathy's acceptance by society and demand from some clients should outweigh these negatives in the considerations of the profession's regulator. A 2016 petition to the RCVS asking for a ban on the homeopathic treatment of animals by veterinary surgeons (Chambers 2016) was

signed by over 1,000 veterinary surgeons, and in light of that, and of changes to the way homeopathy is viewed by the NHS, and other factors, at the time of writing the RCVS is reviewing its position and guidance on the use of complementary and alternative medicines by veterinary surgeons (Royal College of Veterinary Surgeons 2016, Anon 2017, Whitehead and others 2017).

REFERENCES

*ANON (2017) RCVS Council overwhelmingly rejects position statement on CAM therapies. **Veterinary Record** **180**:602*

AUSTRALIAN GOVERNMENT (2015) National Health and Medical Research Council statement on homeopathy and NHMRC information paper - evidence on the effectiveness of homeopathy for treating health conditions. https://www.nhmrc.gov.au/guidelines-publications/cam02. Accessed March 24, 2017

*CHAMBERS, D. (2016) Petition was aimed at RCVS, not homeopaths. **Veterinary Times.** January 18 edn. p 27*

CONNECTICUT VETERINARY MEDICAL ASSOCIATION (2013) Addendum #1 to CVMA Proposed AVMA Resolution: "Homeopathy has been identified as an ineffective practice and its use is discouraged." White Paper: The Case Against Homeopathy. http://skeptvet.com/Blog/wp-content/uploads/2012/12/Resolution3_2013_Homeopathy_Attch1.pdf. Accessed August 11, 2017

*FEDERAL TRADE COMMISSION (2016) Enforcement Policy Statement on Marketing Claims for OTC Homeopathic Drugs. In: Federal Register **81(239)**, 90122. https://www.ftc.gov/policy/federal-register-notices/federal-trade-commission-enforcement-policy-statement-marketing. Accessed August 11, 2017*

HOUSE OF COMMONS (2010) House of Commons Science and Technology Committee, Evidence check 2: Homoeopathy. www.publications.parliament.uk/pa/cm200910/cmselect/cmsctech/45/4504.htm

NHS DIGITAL (2017) Prescription cost analysis England 2016 http://www.content.digital.nhs.uk/catalogue/PUB23631. Accessed August 11, 2017

NHS ENGLAND (2015) NHS Choices: Homeopathy. http://www.nhs.uk/Conditions/homeopathy/Pages/Introduction.aspx#evidence. Accessed August 11, 2017

NHS ENGLAND (2017) Items which should not be routinely prescribed in primary care: A Consultation on guidance for CCGs.

https://www.engage.england.nhs.uk/consultation/items-routinely-prescribed/. Accessed August 11, 2017

ROYAL COLLEGE OF VETERINARY SURGEONS (2016) http://www.rcvs.org.uk/news-and-events/news/standards-committee-to-review-complementary-and-alternative/. Accessed June 10, 2017

*WHITEHEAD, M.L., PRICE, A., JESSOP, M., GOUGH, A., TAYLOR, N., ATKINSON, M., HYDE, P., CHAMBERS, D., McKENZIE, B. & GUTHRIE, A. (2017) Complementary and alternative medicines. **Veterinary Record** **181**:24*
